# Wavelength multiplexing infrared metasurfaces for protein recognition and trace detection

**DOI:** 10.1515/nanoph-2023-0517

**Published:** 2023-09-26

**Authors:** Shiqing Dong, Chao Dong, Kesheng Shen, Yun Zheng, Jie Sun, Cheng Zhen, Haiyang Hu, Feng Zhang, Zhe Zhang, Hongchao Liu, Hai Lu

**Affiliations:** Engineering Laboratory for Optoelectronic Technology and Advanced Manufacturing, School of Physics, Henan Normal University, Xinxiang 453007, China; Henan Key Laboratory of Optoelectronic Sensing Integrated Application, College of Electronic and Electrical Engineering, Henan Normal University, Xinxiang 453007, China

**Keywords:** metasurface, wavelength multiplexed, refractive index sensing, surface enhanced infrared absorption spectrum

## Abstract

Infrared metasurfaces have exhibited exceptional optical properties that differ from naturally occurring metallic and dielectric nanostructure, enabling non-destructive and label-free sensing in a broadband region. However, implementing wavelength multiplexing sensors in broadband infrared has faced significant challenges. These challenges arise from the difficulty in efficiently exciting high *Q* resonances at specific wavelengths and the inability to individually tune each resonance. Herein, we present a dual resonant metasurface that utilizes a metal–dielectric–metal plasmonic grating and a dielectric–metal channel. By adjusting the vertical and horizontal structures of metasurface, we can independently modify the spectrum of the metasurface in the near-infrared and mid-infrared regions. This broadband infrared metasurface exhibits robust spectral regulation, enabling a polarization-dependent strategy for the dual-resonance. It offers a competitive advantage over traditional metallic nanostructure in refractive index sensing at the second near-infrared window and ultrasensitive vibrational spectroscopy in mid-infrared. Specifically, our proposed metasurface achieves protein concentration sensing and dynamic monitoring of protein concentration in the infrared two-zone. Additionally, it enhances the mid-infrared absorption of amide II with a high *Q* resonance. The metasurface which combines wavelength multiplexing and polarization dependent switch for protein recognition and trace detection, presents a novel approach for developing high-performance sensors and Integrated photonics sensors in the broadband infrared region.

## 1 Introduction

The abundant photon degrees of freedom, including the wavelength, polarization, amplitude, phase, and time, can be modulated by the optical metasurface with the well-designed subwavelength structure for the detection of multi-dimensional biochemical information [1, 2]. Among them, the diversified biological information can be obtained at a broadband wavelength from visible to terahertz [3–5]. The second near-infrared (1000 nm–1700 nm) is particularly advantageous for the spectroscopy and imaging *in vivo* due to its high penetration depth, low absorption, and scattering [6]. By tracing blood flow with high spatial and temporal resolution can detect the biological processes in the brain at the molecular scale [7, 8]. Additionally, the protein recognition and conformational analysis rely on detecting vibrational fingerprints in the mid-infrared, which reflect the protein secondary structure and identify functional groups. Recently, the surface enhanced infrared absorption spectrum (SEIRA) based on optical metasurface has been proposed as a method to amplify the conformational characteristics of molecules [9–13]. Hence, developing wavelength multiplexing metasurfaces in the broadband region will provide the cross validation of protein sensing for research and diagnostics, enabling small-volume, real-time, label-free molecular recognition from the various protein species [14–17]. However, achieving wavelength multiplexed detection on a target molecule is still challenging due to the material limitations and crosstalk between the different modes of structural resonance [18, 19].

Metallic nanostructure has been currently explored in a broadband spectral region across different wavelength ranges, from terahertz to ultraviolet [1, 2, 16, 17, 20, 21]. These nanostructures have the unique ability to confine and control the free-space propagating light into subwavelength volumes, resulting in extreme enhancements of electromagnetic fields. Recent studies have used log-periodic trapezoidal and hook nanoantennas to achieve multispectral SEIRA spectroscopy [22–24]. However, the spectral resonance and figure of merit of nanostructure are constrained by the multiscale geometries and intrinsic Ohmic losses in noble metals, respectively [25–27]. On the other hand, all-dielectric metamaterials have been highlighted for SEIRA spectroscopy, specifically in achieving high-*Q* resonance narrower than the target molecular vibrational bands [13, 28–31]. This enables monochromatic SEIRA sensing without the need for an IR spectrometer. However, compared to antennas made from precious metals, all-dielectric antennas are challenging to chemically modify for enhancing their biological affinity [32–34].

Artificial metamaterials composed of both horizontal and vertical subwavelength structures have shown extraordinary broadband spectral modulation performances that differ from naturally available materials [35, 36]. The scale difference between vertical and horizontal structures is usually above two orders of magnitude, resulting in the resonance covering a wider wavelength range [37, 38]. Specifically, metamaterials can tune the resonance in high frequency through the nanometer horizontal films and achieve high-*Q* perfect absorption through vertically stacked metal–dielectric–metal (MDM) layers [39, 40]. This has led to exciting possibilities for designing the wavelength multiplexing sensor with the high-*Q* resonance [41, 42]. However, achieving high-*Q* resonant wavelength multiplexing over a broadband infrared spectrum remains a challenge. That is, the single high-*Q* nanoantenna geometry has limited light enhancement ability within a narrow bandwidth. The large scaled structure array periodicities can produce the multi-order modes that expand the spectral range to the near-infrared, but these high-frequency modes are typically weak and disturb the line shape of high-frequency spectrum [43]. Additionally, the most metasurface designs remain challenging to accommodate resonant modes with equally strong excitation efficiencies over a broad spectrum [44]. This generally requires precise and costly micro/nano fabrication techniques [45, 46].

**Figure 1:**
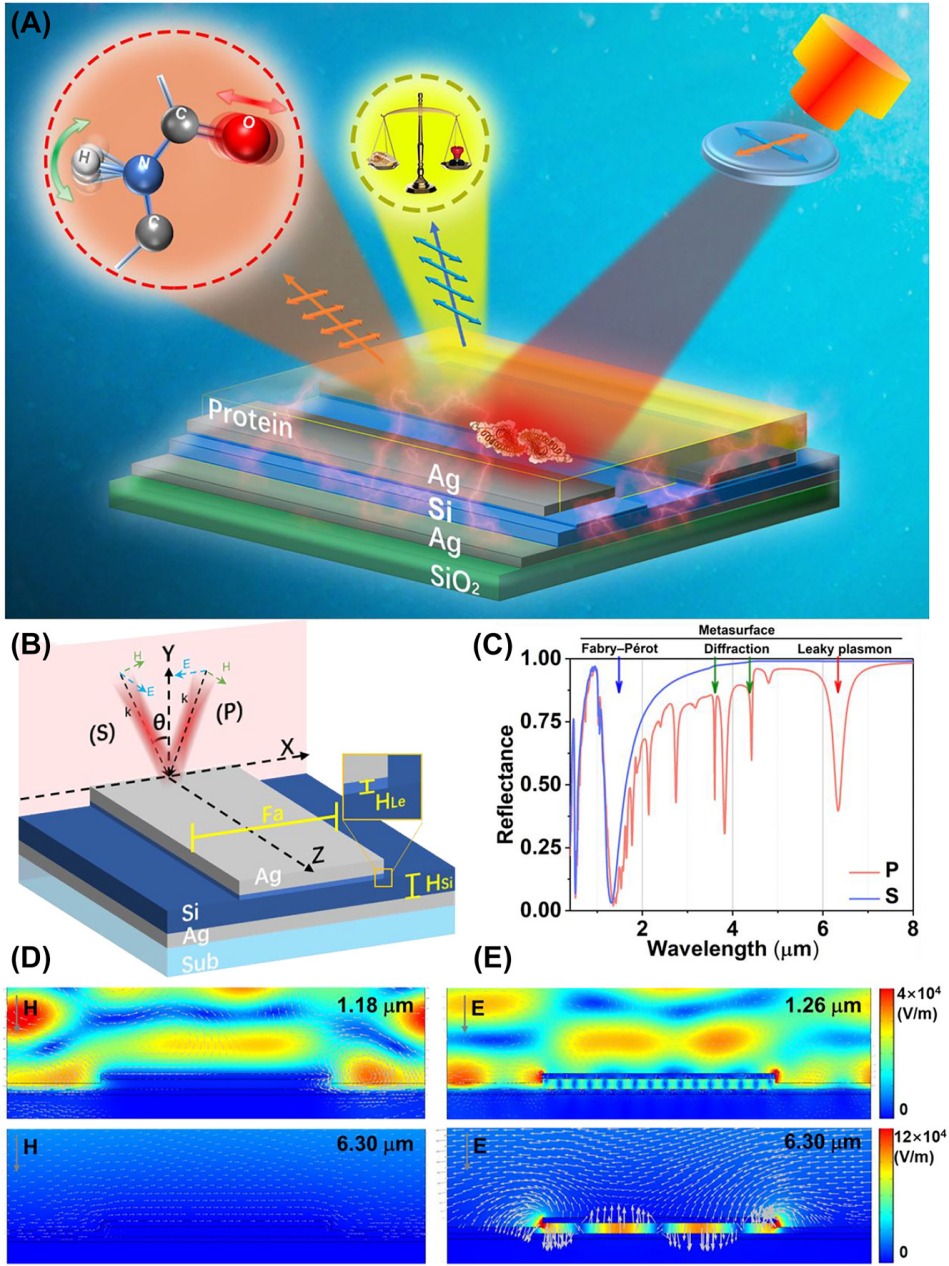
The overview diagram of wavelength multiplexing infrared metasurfaces for protein recognition and trace detection. (A) The schematic of metasurface for protein sensing. (B) The schematic of a layered metal (Ag)–dielectric (Si)–metal (Ag) subwavelength-scale structure on a substrate (SiO_2_), (C) the reflectance of metasurfaces with the S and P polarization with 6° incidence. (D) The electric field distributions and the magnetic vector of S polarization with 6° incidence. (E) The electric field distributions and electric vector of P polarization with 6° incidence.

In this study, we propose a dual resonant metasurface based on the MDM plasmonic grating and dielectric–metal (DM) channel for wavelength multiplexing infrared protein sensing. The collaborative spectral regulation in both horizontal and vertical dimensions offers advantages over antenna regulation based solely on geometric structure. The thin film interference produces a polarization-insensitive resonance in the infrared two zone, which can be used for refractive index (RI) sensing. The MDM grating-like metasurface produces a polarization-dependent high-*Q* resonance in the mid-infrared, which can be used for SEIRA to identify the amide II of bovine serum albumin (BSA). The metasurface spectrum demonstrates strong robustness regulated by polarization. Our experiments highlight the effectiveness of wavelength multiplexing metasurface as a versatile platform for accessing the mass and conformational information of protein in a sensitive and reliable manner, even in cases of trace protein deposition (Figure 1A). This plasmonic biosensor holds great promise for providing fundamental knowledge on changes in protein conformations, including ultrasensitive dynamic diagnostics and high-information analysis of molecular reactions.

## 2 Results and discussion

### 2.1 The infrared spectral design of metasurface

Figure 1B illustrates the configuration of metasurfaces that integrate a vertical MDM sandwich structure with a horizontal grating-like subwavelength structure. The bottom Ag film (60 nm) acts as a mirror, exhibiting high reflectance across the entire infrared band. The middle Si film (100 nm), characterized with a high RI and low infrared losses, is sandwiched between two Ag films, thus creating a subwavelength Fabry–Pérot cavity that enhances resonance. By employing low loss dielectric materials, the resonant energy is efficiently absorbed by the sensing material instead of being lost within the structure itself. Subsequently, a grating-like patterning is etched onto the sandwich structure. The metasurface is determined with the following structural parameters: the width of top Ag film (Fa = 2.2 μm), cycle (4 μm), the remaining height of the Si film after etching (*H*_Si_ = 80 nm), and the etching depth of Si film (*H*_Le_ = 20 nm). The incident plane is perpendicular to the channel direction of the grating, indicating as a red plane in Figure 1B. The senkrecht (S) polarization is defined with the electric vector paralleling to the *z* direction. Meanwhile, the parallel (P) polarization is defined with the electric vector paralleling to the *x* direction.

Figure 1C depicts the reflectance spectrum across a range of 0.36 μm–8.00 μm with normal incidence under different polarizations. The related spectrum under S polarization reveals two resonances at 0.48 μm and 1.18 μm, indicating that only the Fabry–Pérot modes produced by Si and Ag films is excited. Meanwhile, the spectrum under P polarization not only excites the resonances at 0.52 μm and 1.26 μm, but also triggers the diffraction and leaky plasmon modes of metasurface in mid-infrared. To distinguish the two modes mentioned above, we studied the response of spectra to incident angles (Figure S1). The diffraction resonance at 4.03 μm was split into two resonance peaks with the increased incident angle, while the leaky plasmon resonance is not sensitive to the varying of incident angle. The based mode of leaky plasmon is located at 6.30 μm. The *Q*-factor of leaky plasmon mode is calculated as 23.78. The rest resonances are the higher modes of leaky plasmon modes. Above all, the fabrication process gives rise to the triple distinct optical models: the Fabry–Pérot mode based on the DM thin film, the diffraction mode of metasurface and the leaky plasmon mode based on the MDM waveguide. These results indicate that the structural spectrum significantly relies on polarization. It demonstrates the robustness of metasurface resonance. According to the actual experiment measurements, the incident angles in both the experimental and numerical simulation reflection spectra are set as 6° in this article.

Figure 1D illustrates the norm electric field and the electromagnetic vectors of resonances corresponding to S polarization with 6° incidence, aiming to provide further insights into the potential physical mechanisms. It is observed that the electric fields at wavelengths 1.18 μm are primarily localized within the channel of metasurface above the Si film. These fields are characterized by Fabry–Pérot mode resulting from interference between a silicon film and Ag film, independent with the polarization. The magnetic field vortex above the grating channel is generated by the combination of oblique incident light and the ridge of top Ag film. Notably, the resonance occurring at 0.48 μm represents a higher mode in comparison to the base mode seen at 1.18 μm (Figure S2A). Due to the interaction between incident and reflected light, the electric field intensity can be enhanced by the Fabry–Pérot mode. Consequently, these findings indicate the metasurface possesses a high sensitivity to the RI of deposition on its surface. Additionally, the spectrum under S polarization has no resonances in mid infrared.

The norm electric field and electric vector of resonances under P polarization with a 6° incidence are demonstrated in Figure 1E. The resonance at 1.26 μm is not only mainly produced by Fabry–Pérot mode, but also accompanied with the crosstalk from the higher-order leaky plasmon modes in MDM structure. Meanwhile, the losses of metasurfaces in mid-infrared are effectively reduced by incorporating a Si film, resulting in a high *Q* resonance. The incident electromagnetic energy is coupled within the middle Si film. There is energy leakage at the edge of top Ag film, creating a leaky plasmon resonance at 6.30 μm. It can further enhance the absorption of surrounding substances. The high *Q* resonance observed at 3.80 μm corresponds to the higher-order modes resonance determined by the Fa (Figure S2B). Furthermore, the green arrow in the figure indicates the direction of the electric vectors. The antiparallel currents at 6.30 μm are induced between the two Ag films, thereby generating a magnetic dipole. The magnetic dipole causes the magnetic field of the reflected radiation to be opposite to that of the incident light, resulting in both electric and magnetic resonance of the metasurfaces in this configuration [47, 48]. The leaky plasmon resonance confines a significant amount of electromagnetic energy in the intermediate spacer layer and leaked the energy above the metasurface, leading to higher spectral absorption and enhanced signal strength compared to pure electric resonance.

**Figure 2:**
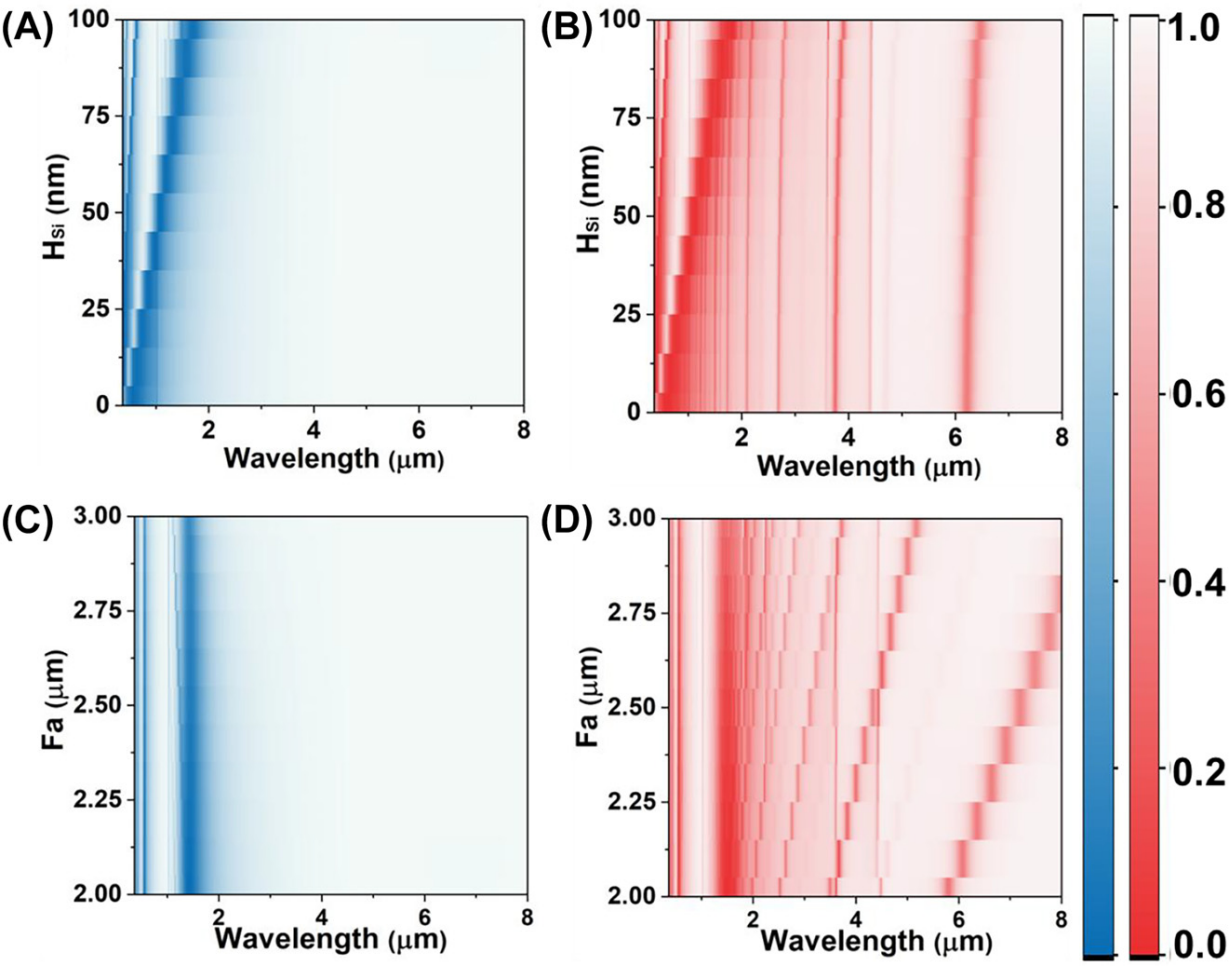
Dependence of the numerical reflection spectra on different geometric structure with the Fa from 2 to 3 μm under the S polarization (A) and the P polarization (B), the thickness of remaining Si film from 0 to 100 nm under the S polarization (C) and the P polarization (D).

Based on the above physical mechanism, the resonance of metasurfaces can be modified separately by adjusting Fa and *H*_si_ depicted in Figure 2. The Fabry–Pérot resonance in the near-infrared is achieved through the interference between the Si films and the bottom Ag mirror. As the *H*_Si_ increases, the resonance wavelength is shifted from the visible to the near-infrared (Figure 2A). Meanwhile, the spectra under P polarization also show the Fabry–Pérot mode resonance determined by *H*_Si_ is insensitive to the polarization (Figure 2B). But the line shapes of spectra are interfered with the high-order leaky plasmon resonances. Additionally, the dual resonance in mid-infrared is also insensitive to the varying of *H*_Si_, demonstrating the robustness of metasurface spectrum. Herein, the thickness of Si film was set as 80 nm to ensure that the feature peak changes remain within the second near-infrared window of human body.

Figure 2C shows the varying Fa has no impact on the resonances produced by the Fabry–Pérot mode resonance in near infrared under the S polarization. Meanwhile, under the P polarization, the Fabry–Pérot resonances in near-infrared are also desensitized to the Fa (Figure 2D). But the leaky plasmon resonances in mid-infrared experience a red-shift from 5.8 μm to 8.0 μm with the increased Fa. To efficiently achieve the SEIRA, the resonant metasurfaces interacts with the molecular vibration at a matched wavelength with the plasmon–phonon coupling, enhancing the fingerprint absorption. Namely, the Fa is set as 2.2 μm to ensure that the absorption peak coincides with the amide absorption of the protein (6.0 μm–6.5 μm).

Moreover, the function between *H*_Le_ and reflectance spectrum under S and P polarization are demonstrated in Figure S3. The leaky plasmon resonances under P polarization are blue-shifted with the increased *H*_Le_. And the reflectance is decrease to 0.02, indicating the enhancement of absorption. Hence, the *H*_Le_ is set as 20 nm to ensure that the resonance wavelength at 6.30 μm is coincided with the absorption of the amide. These results highlight the ability of the horizontal and vertical structures of the metasurface to independently adjust the dual-resonances in the near infrared and mid infrared. And the resonances can be switched by changing the polarization. These properties of metasurface contribute to the robustness of the spectrum.

### 2.2 The numerical protein sensing of metasurfaces

**Figure 3:**
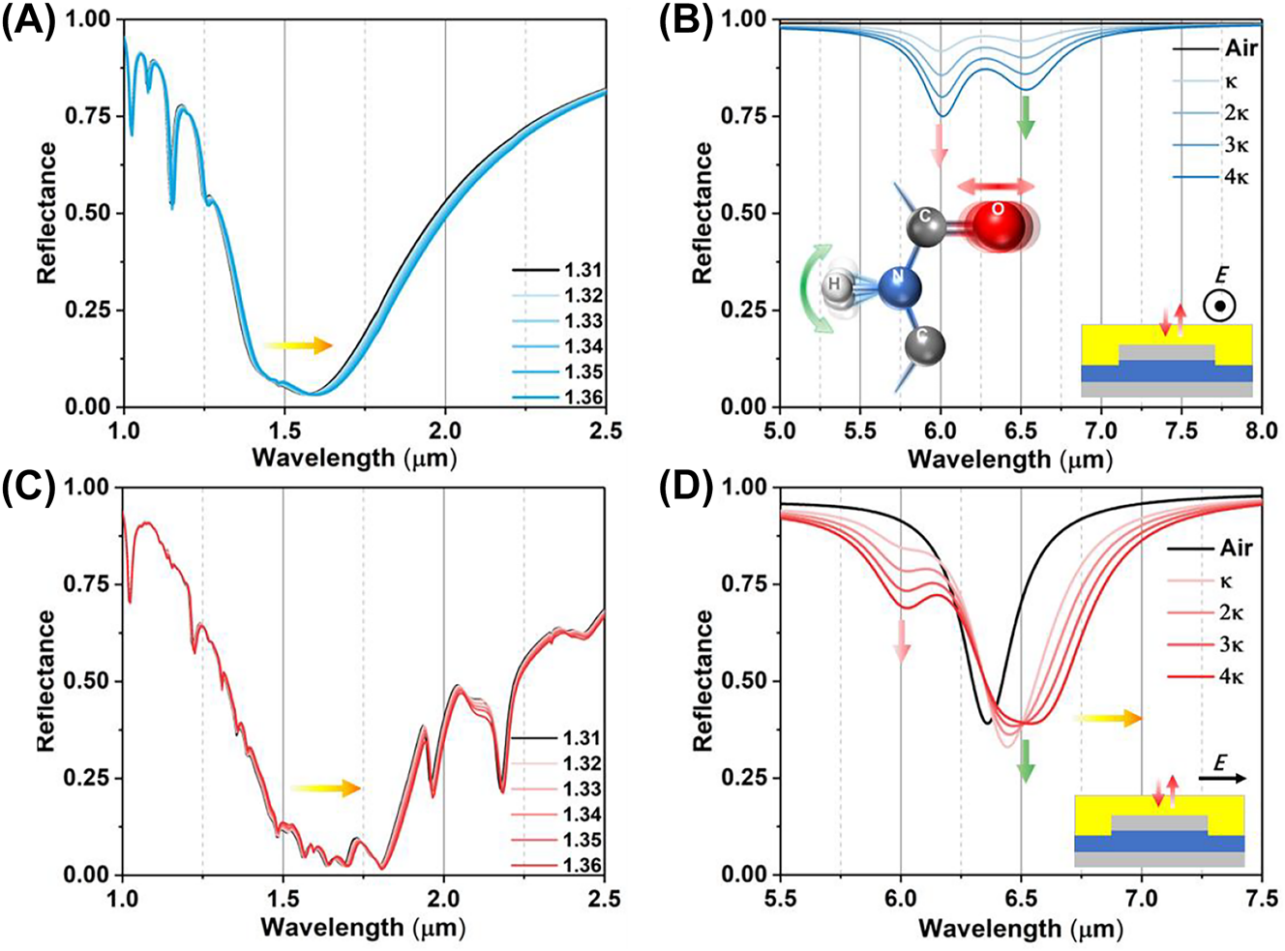
The numerical reflection spectra with functional protein coating. The reflectance in near infrared (A) and mid infrared (B), the schematic inset illustrates the plasmonic internal reflection measurement configuration under the S polarization. The reflectance in near infrared (C) and mid infrared (D). The schematic inset illustrates the plasmonic internal reflection measurement configuration under the P polarization.

**Figure 4:**
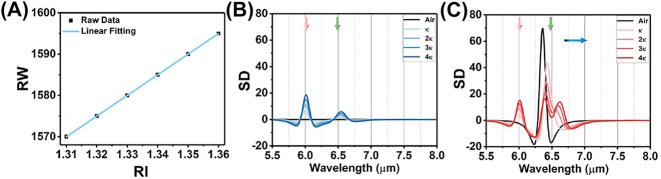
The analyses of reflection spectra with functional protein coating. (A) The RI sensitivity in near infrared under S polarization defined by the linear fitting between resonance wavelength (RW) and RI. (B) The second derivative (SD) of reflection spectrum with S polarization in mid infrared. (C) The SD of reflection spectrum with P polarization in mid infrared.

To further demonstrate the protein sensing ability of wave-length multiplexing metasurfaces, we conduct a numerical experiment to observe how the deposition of protein film on the top of metasurfaces affects their reflection spectra in Figure 3. We introduce a 400 nm protein dispersion block, represented by a Lorentz model with two coupled oscillators (Section 3.1) on the metasurface. The density of protein deposition is strongly related with the RI of protein, which can be monitored by the polarization insensitive resonances of metasurfaces in near infrared. Both reflectance spectra under S and P polarization are red-shifted with the RI of protein (Figure 3A and C). However, the P-polarized spectrum in near infrared is susceptible to interference by the high-order modes of leaky plasmon resonances. To overcome this issue, we will use S-polarized spectrum to achieve protein concentration sensing in following studies. The RI sensitive under S polarization is achieved at 500 nm/RIU (Figure 4A).

The absorption of protein at 6.00 μm and 6.47 μm is observed in the reflection spectra under S polarization, indicating the C=O stretching vibration of amide I and the N–H bending vibration of amide II (Figure 3B). The reflect spectra are similar with the one obtained from protein coverage on a silver mirror. As the protein deposition on the metasurfaces, the reflectance is proportionally decreased with the protein extinction coefficient (*κ*). It is further confirmed by the second derivative (SD) of the reflection spectrum. The degree of spectral depression correlates with protein absorption (Figure 4B). Meanwhile, the leaky plasmon resonance with P polarization at 6.30 μm alters the line shape of the reflectance spectrum in two ways (Figure 3D): (i) the leaky plasmon resonance was red-shifted with increasing the *κ* of protein. (ii) The absorption of amide II is enhanced when the metasurface resonance is overlapped with the absorption of amide I. These features are further evident by the SD of the reflection spectrum (Figure 4C). It is important to note that the absorption of amide II is not directly proportional to the protein concentration. In summary, these numerical simulations suggest that the metasurface can potentially achieve protein recognition and trace detection in a broadband infrared range by switching the polarization.

### 2.3 Fabrication and characterization of metasurfaces

**Figure 5:**
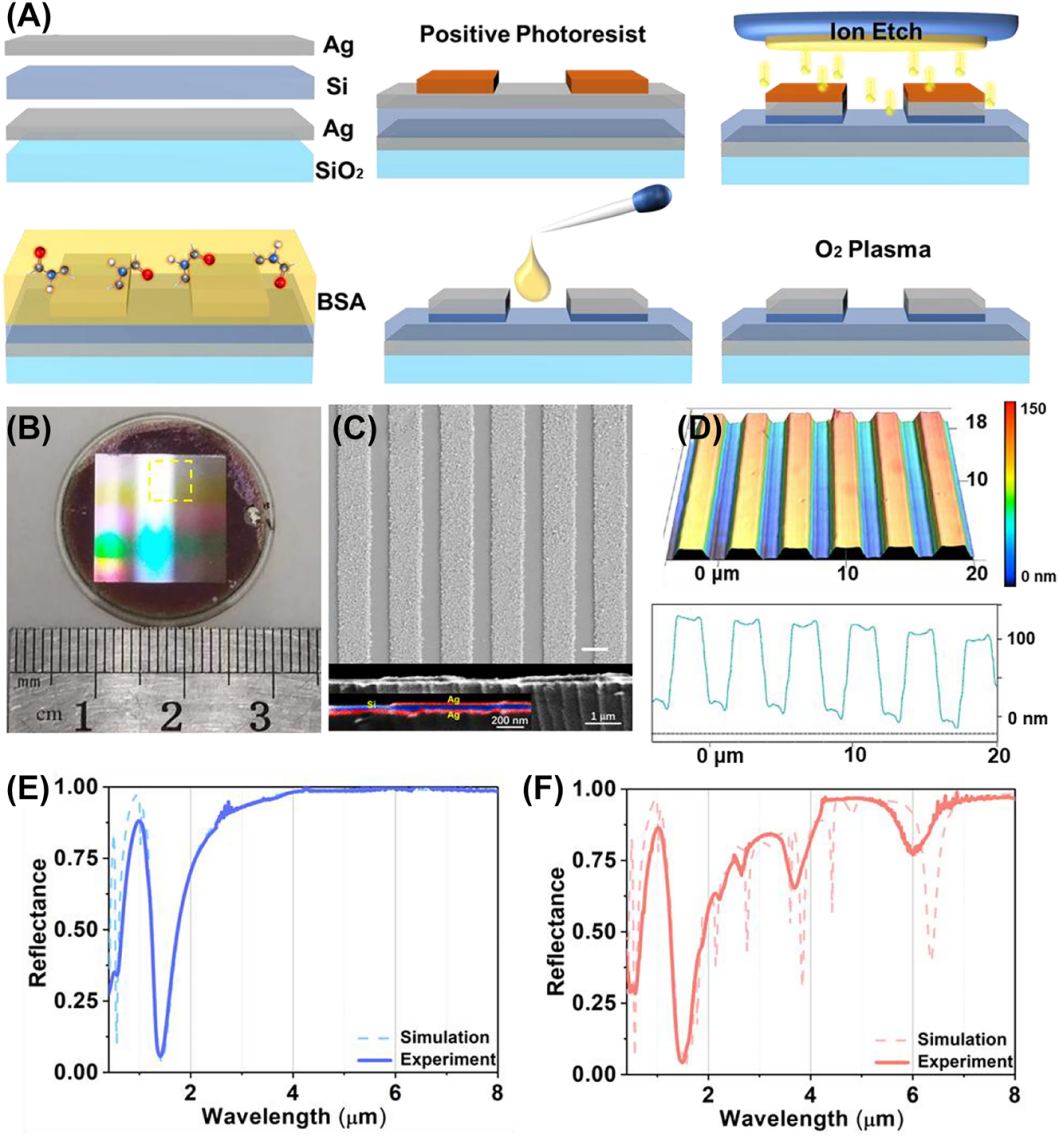
The optical characterization of metasurfaces. (A) Schematic of the fabrication process. (B) The photograph of metasurface sample with diameter of 2.5 cm in visible light. (C) The top view and cutaway of metasurface with SEM. (D) the metasurface morphology of confocal microscope, the vertical structure shown in the diagram is 10 times larger than its original size. The full spectral range spectrum under S (E) and P (F) polarization from 0.36 μm to 8.0 μm with 6° incidence.

In this section, we conduct further experiments to validate the numerical results obtained from the designed metasurface sensor. The fabrication process of the metasurface is illustrated in Figure 5A (more details in Section 3.2). The photograph of the metasurfaces demonstrates the rainbow stripes generated by thin film interference in the visible range, indicating that the different conditions of interference are matched by inhomogeneous spatial structural parameters leads to iridescent phenomenon (Figure 5B). The high reflectivity region corresponds to the metasurface area, which is formed by the top Ag layer. The black area on the edge of substrate represents the exposed silicon film after etching. The morphology of the metasurface geometry is characterized by scanning electron microscopy (Figure 5C). The metasurface are uniformly distributed with a graininess surface and consistent periodic length. The inhomogeneous surface at Ag film is located at the ridge of metasurface. Moreover, Figure S4 shows the inhomogeneity surface has a limited impact on reflectance spectrum. The wavelength of each resonance is not shifted by the inhomogeneous surface at Ag film, but the reflectance of resonances is slightly decreased.

The vertical structure consist of top Ag, middle Si and bottom Ag layers are approximately 60 nm, 100 nm and 60 nm, respectively, while the grating width is approximately 2.2 μm. The scale difference between the horizontal and vertical structures of metasurfaces is larger than one order of magnitude, promoting the corresponding resonance distribution in the broadband infrared spectrum (Figure S5). The thickness of each layer is determined by using spectroscopic ellipsometry on the accompanying substrates. The confocal microscopy shows the ion beam etching depth from the top of the metasurface is measured to be 100 nm (Figure 5D). The morphology image after removing the top Ag film through ultrasound shows that the grating protrusion is composed of two layers: Si and Ag film (Figure S6). And the thickness of top Ag film is measured as 60 nm, which is consistent with the measurement of elliptical polarization spectrometer. These structural data indicate that the metasurface sensor is successfully constructed according to the theoretical parameters.

The optical properties of the metasurface are investigated by measuring the spectral reflectance with different polarizations. The spectral measurements in the broadband infrared range are measured by using the Cary 5000 UV-Vis-NIR spectrophotometers (0.36 μm–2.5 μm) and the FTIR spectrometer (2.5 μm–8.0 μm) equipped with a reflective attachment under S and P polarization. Under S polarization, the measured spectrum exhibits two resonances in the visible and near-infrared regions. The simulated spectrum (dash line) closely matches the measured spectrum (Figure 5E), indicating that the Si layer thickness remain at 80 nm, as shown in Figure 2A. Similarly, the simulated spectrum under P polarization perfectly matches the measured spectrum in the broadband infrared range (Figure 5F). However, due to nonuniformity and defects of fabrication (e.g., trapezoidal shape, edge sharpness, and ribbon size deviations), the line shape of the simulated leaky plasmon resonances is shaper than that observed in the experiment. These results demonstrate the consistency between the theoretical predictions and experimental measurement.

### 2.4 Multiplexing infrared sensing for protein

**Figure 6:**
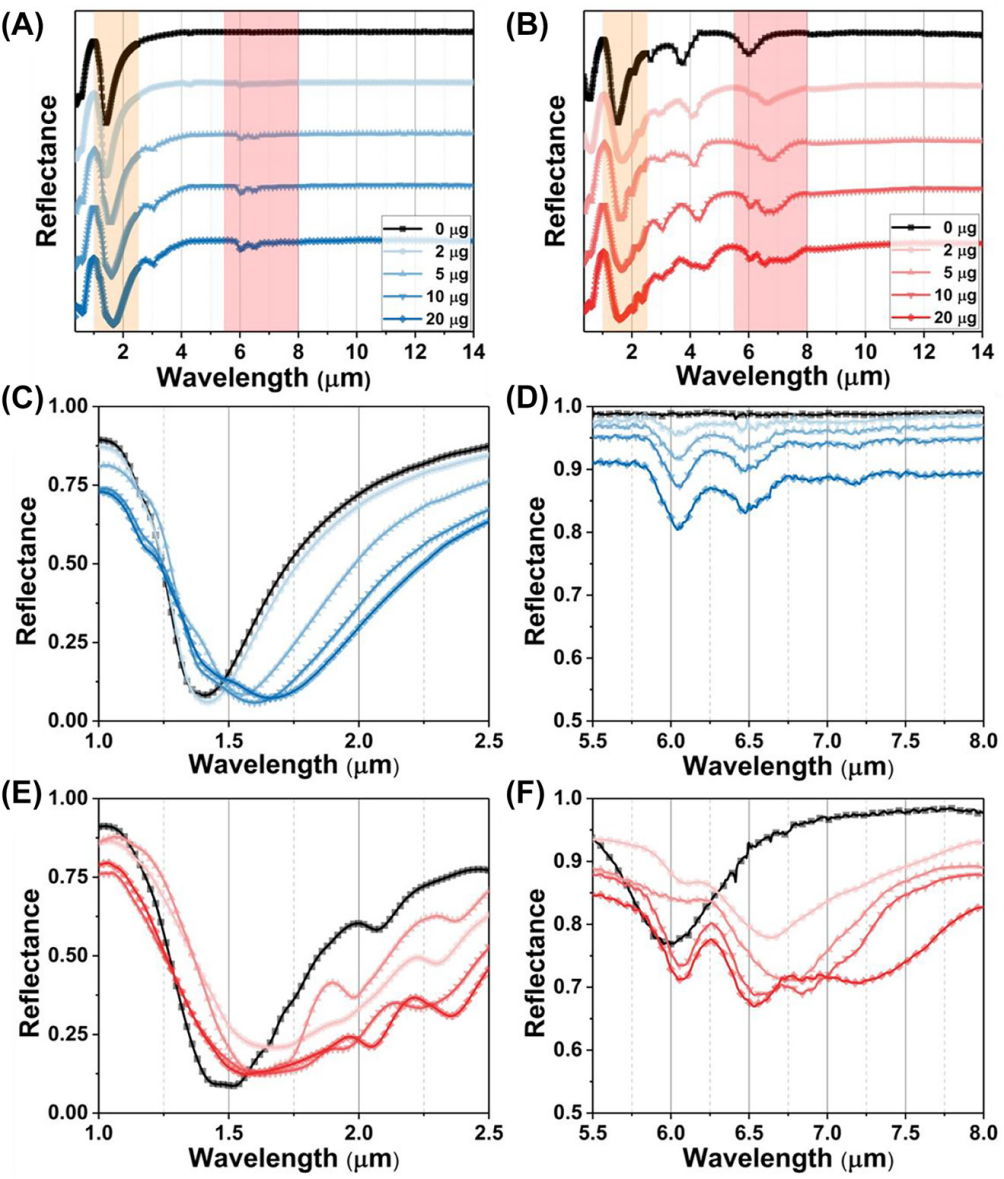
The cooperative RI and SEIRA protein sensing for BSA. (A) The broadband spectrum under S polarization. (B) The broadband spectrum under P polarization. (C) The RI sensing for BSA with the S polarization. (D) The infrared spectra for BSA under the S polarization. (E) The RI sensing for BSA with the P polarization. (F) The SEIRA sensing for BSA with the P polarization.

**Figure 7:**
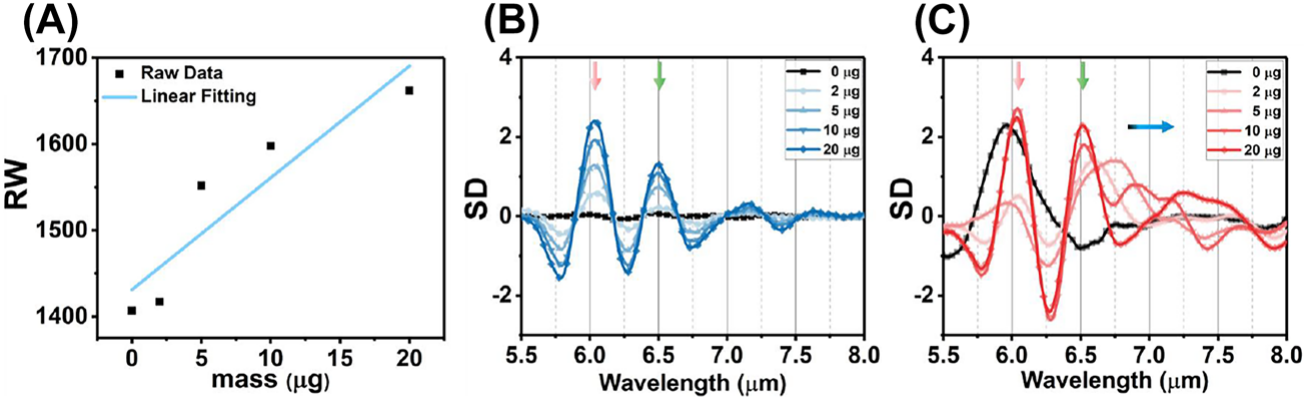
The analyses of reflection spectra with BSA coating. (A) The RI sensitivity in near infrared under S polarization defined by the linear fitting between RW and BSA mass. (B) The SD of reflection spectrum with S polarization in mid infrared. (C) The SD of reflection spectrum with P polarization in mid infrared.

For the experimental validation of the wavelength multiplexing sensing, the BSA is used as a model protein for demonstrating the RI and SEIRA protein sensing. Figure 6A depicts the experimental broadband reflection spectra under S polarization before (black) and after (blue) coating with varying masses of BSA. Increasing the mass of BSA leads to a spectral redshift in the near infrared region (Figure 6C), indicating the increased RI around the metasurface. The RI sensitivity is calculated as 12.96 nm/μg by linearly fitting the relationship between protein mass and RW (Figure 7A). The mid-infrared spectrum shows the absorption of amide I (6.054 μm) and amide II (6.495 μm) are proportionally increased with BSA mass (Figure 6D). The SD of spectrum also further analysed gain insights into the secondary structure of protein (Figure 7B). Additionally, we used multiple peak fitting to obtain information on the center wavelength and full-width at half-maximum (FWHM) of amide absorption for further analysis (Supplementary Table 1). The results indicate that protein deposition does not significantly alter the configuration of protein, but proportionally enhances the absorption of amides in mid-infrared.

Figure 6B show the measured reflection spectra under P polarization before (black) and after (red) coating a series mass of BSA. The spectra in near infrared are redshifted with the BSA coating (Figure 6E). However, the FWHM of resonance in near infrared is broad owing to crosstalk from the higher-order leaky plasmon modes, which is not suitable for RI sensing. Meanwhile, the spectrum in mid infrared includes the absorption of amide at 6.054 μm and 6.495 μm, as well as the metasurface resonance (Figure 6F). The overlapping of three resonances poses a challenge in distinguishing spectral signals accurately. To analyze the constituting spectral components, we utilized the SD of the spectrum to determine the wavelengths of three absorption information (Figure 7C). The results demonstrate that the accumulation of proteins does not cause any significant shift in the wavelength of amides absorption (red and green arrow). However, the resonances, red-shifting from 6.789 μm to 7.147 μm, indicate that the leaky plasmon resonances are also highly sensitive to RI caused by protein deposition (blue arrow). Furthermore, it is noteworthy that the FWHM of BSA absorption is coincides with that of metasurface resonances. The following results will show the resonance with high *Q*-factor exhibit varying levels of enhancement in the absorption of amide I and amide II.

Previous research has indicated that the vibrational signals in SEIRA spectra arise from the coupling between the broad resonance of *ω*_res_ and the narrow vibrational mode of *ω*_vib_, resulting in an asymmetric Fano-like profile [49]. To analyze the molecular infrared absorption, the AsLSS algorithm is employed to calculate the baseline, which is then compared to the original spectrum to determine the spectral difference. However, the AsLSS algorithm is primarily effective for sharp and small peaks. Difficulties arise when the high *Q* resonance of *ω*_res_ closely resembles the molecular absorption of *ω*_vib_, making it challenging to accurately obtain the baseline using the above algorithm. Therefore, we propose the utilization of the multi-peak Lorentz model with fixed parameters to precisely quantify the infrared signals of both molecular absorption and surface resonances.

The spectral fitting and extraction of physical parameters under S polarization are conducted by determining the dual Lorentz absorption of BSA. Firstly, we obtain the FWHM and wavelength of amide I and amide II through dual-peak Lorentz fitting under S polarization (Figure S6). The C=O stretching vibration of amide I is located at 6.049 μm (1653 cm^−1^) with an FWHM of 210 nm. The N–H bending vibration of amide II is found at 6.452 μm (1550 cm^−1^) with an FWHM of 300 nm. Additionally, the absorption of amides is analyzed by calculating the area of each Lorentz peak. The areas of amide absorption are linearly increased with the protein mass (Supplementary Table 1). And the absorption ratios between amide II and amide I are consistently maintain at around 88 % with the deposition of BSA on the metasurfaces (Figure 9A, red bar). These results indicate that the absorption of both amide I and amide II improved proportionally with the increase in mass. Furthermore, under S polarization, the metasurfaces cannot enhance the absorption of either amide. These parameters are used in subsequent fitting to investigate the specific absorption enhancement of amide II under P polarization.

The spectrum of metasurface without BSA deposition under P polarization can be expressed as a single Lorentz model (Figure S7). The wavelength and FWHM of the leaky plasmon resonance are also determined to be 6.025 μm and 695 nm, respectively. The FWHM of resonance is maintained as a constant in following fitting. Meanwhile, the wavelength and FWHM of amides absorption are also kept constant during fitting. Therefore, the multipeak fitting of reflection spectra with BSA deposition under P polarization can be demonstrated as a triple Lorentz resonant model, including the leaky plasmon resonance of metasurface and the dual-peak absorption of amide.

**Figure 8:**
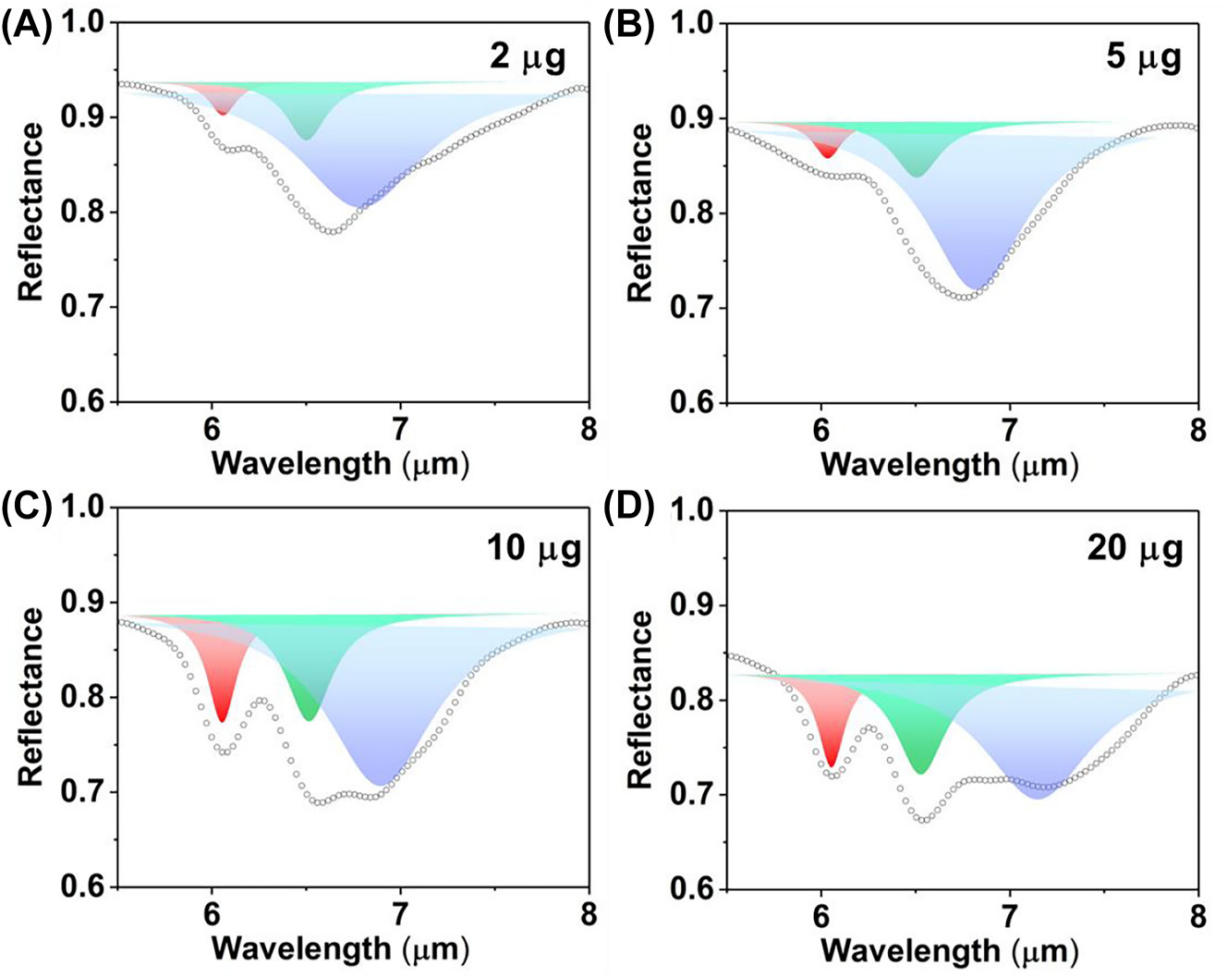
The multipeak fitting of reflection spectra with different mass BSA coating under P polarization (A. 2 μg, B. 5 μg, C. 10 μg, D. 20 μg). The red, green and blue peaks represent the Lorentz peaks of amide I, amide II and metasurface, respectively.

**Figure 9:**
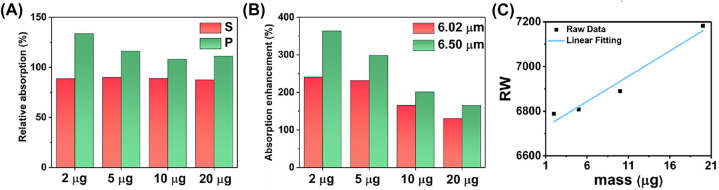
The SEIRA analysing of reflection spectra under P polarization (A) the relative absorption between the amide II and amide I under S and P polarization, respectively. (B) The absorption enhancement defined by the ratio the absorption of amides under P polarization to that of amides under S polarization, respectively. (C) The RI sensitivity of leaky plasmon resonance with P polarization in mid infrared defined by the linear fitting between RW and BSA mass.

The triple Lorentz fitting of P-polarized spectra accurately represents the resonance of the metasurface for RI sensing and the absorption of amides for SEIRA analysis (Figure 8). The resonances of the metasurface are depicted as blue Lorentz peaks. The wavelengths are red-shifted from 6.789 μm to 7.147 μm with the BSA deposition (Supplementary Table 2), with an RI sensitivity of approximately 22 nm/μg (Figure 9C). Although the plasmon resonance in P-polarization has higher refractive index sensitivity than the Fabry–Pérot resonance in near-infrared, the mid-infrared plasmon resonance in the P-polarization would be interfered by the absorption of water *in vivo* and/or liquid sample. Moreover, the leaky plasmon resonance is overlapped with the absorptions of amide, requiring additional fitting analysis to obtain the redshift. And the mid-infrared sensor is not as mature as near-infrared detection methods. Hence, the refractive index sensing of near-infrared resonance is more convenient for biological sensing.

The absorption of amide I and amide II are represented by the red and green Lorentz peaks, respectively (Figure 8). The absorption of amide I and amide II under P polarization demonstrated in Supplementary Table 2. The absorption enhancement defined by the ratio the absorption of amides under P polarization to that of amides under S polarization, respectively. The absorption of amide I with 2 μg increases by 241.05 % under P polarization (Figure 9B, red bar). The metasurface resonance (6.789 μm) and amide I absorption (6.058 μm) show the highest degree of overlap at a BSA deposition mass of 2 μg, suggesting a strong interaction between the metasurface and the protein. Meanwhile, the absorption of amide II under P polarization is enhanced by 363.69 % (Figure 9B, green bar). It is the maximum overlap between the resonance peak (6.789 μm) and the amide II absorption peak (6.511 μm).

Because the overlap between the absorption of amide I and resonance are not as good as the overlap between the absorption of amide II and resonance, the absorption of amide I only increases by 241.05 % under P polarization, while the absorption of amide II under P polarization increases by 363.69 %. As the resonance of metasurface undergoes a red shift due to protein deposition, the absorption enhancement of amide I and amide II are gradually decrease to a same level around 150 %. The results indicated that the absorption enhancement reached the maximum when the resonance wavelength of metasurface is consistent with the wavelength of amide absorption. Due to the red-shift of metasurface resonance with the increased BSA deposition, the overlap between metasurface resonance and the absorption amides is reduced, leading the enhancement gradually decreases as the mass increases.

The high *Q* resonance of metasurface also leads to a varying degrees enhancement on infrared absorption of amide I and amide II. It is evident from the ratio of amide II absorption to amide I absorption under P polarization is above 100 %, indicating a specific enhancement of amide II by high *Q* resonance (Figure 9A, green bar). These findings provide valuable insights into the intricate interplay between the metasurface resonance and the absorption characteristics of different amides, thereby shedding light on the potential applications of this metasurface in various sensing and analysis techniques.

## 3 Methods

### 3.1 Metasurface design and simulation

All numerical simulations in this study were performed using the commercial finite element method software COMSOL Multiphysics. To optimize computational efficiency, a two-dimensional optical simulation was implemented, assuming the metasurface have infinite extension in the *Z* direction. The top and bottom boundaries in the *X* direction were set as periodic ports, while perfectly matched layer (PML) boundary conditions were applied. In the *Y* direction, periodic boundary conditions were employed. The optical response of metasurface was analyzed by considering a light beam entering the grating with a specific polarization and angular orientation. To ensure accurate calculations, a fine mesh size of 10 nm was used at the nanostructure interface. The RI and extinction coefficient of Si and Ag films were measured using ellipsometry (VASE VB-400), and compared with our preview study [50]. According to the *Q*-factor expressed in Equation (1):

(1) $$Q=\lambda_{\text{res}}/\text{FWHM}$$

where the *λ*_res_ is the wavelength of resonance. The inevitable manufacturing errors will degrade the performance of the actual sample. There are two types of deformations that occur: rounded corners and pyramid-like deformations. The radius of the rounded corners at the top Ag interface and the Si film is set at 20 nm. For the second type of deformation, a trapeziform grating is used, with the ratio of the top edge to the bottom edge chosen as 0.95. Although the metasurface maintains its properties of ultraweak angular dispersion and high *Q*-factor when these deformations are introduced, the *Q*-factor of the fabricated samples will be reduced due to the resonant wavelength shift caused by the inhomogeneity of the unit cells and polarization sensitivity.

During the numerical protein sensing of metasurfaces, the thickness of coating protein was set as 400 nm. The dielectric constant of protein was defined by the Lorentz model come as follow [51]:

(2) $$\varepsilon_{r}=\varepsilon_{\infty }+\overset{2}{\underset{i=1}{\sum }}\frac{S_{i}^{2}}{\omega_{i}^{2}-\omega^{2}+i\omega \gamma_{i}}$$

where *ɛ*_∞_ is the isotropic high-frequency dielectric constant as 2.08. the *ω*_*i*_ are the frequencies of amide I (1668 cm^−1^) and amide II (1550 cm^−1^). The *γ*_*i*_ are the damping rates of amides (78.1 cm^−1^ and 78.1 cm^−1^). The *S*_*i*_ are oscillation strengths for the amide I (213 cm^−1^) and II (200 cm^−1^) bands, respectively.

### 3.2 Device fabrication

The sandwich films were deposited on the glass substrate by Ion beam sputtering deposition. The thickness of each layer was determined through those of the accompany glass. After each layer was deposited, the accompanying glass was removed and measured to determine the thickness of each film using an elliptical polarization spectrometer (VASE VB-400). The horizontal structure was patterned using the ultraviolet lithography with the positive photoresist (URE-2000/35). Ar gas was used for ion etching to transfer the mask patterns onto the metasurface (MIBE-150). The etching speed in the Ag and Si layers was determined by recording the time it took for the accompanying glass to be completely engraved. The photoresist above the metasurface was soaked in acetone and the unexposed resist was cleaned in a 100 % plasma O_2_ environment. A 10 μL BSA was then dropped onto the metasurface. Finally, the samples were carefully dried using a nitrogen blow to eliminate the effects of water absorption.

### 3.3 Metasurface characterization

The Bruker Vertex-70 V FTIR spectrometer, which was equipped with a Globar infrared source and KBr beam splitter, was used to measure the mid-infrared spectrum (4000 cm^−1^–400 cm^−1^, 4 cm^−1^ resolution). The incident angle and polarization of light were controlled by a reflective spectral accessory. The angle of incident light was adjusted using a symmetrical robotic arm and a spherical mirror. A linear polarizer, positioned along the long axis of the channel, was used in the path of the incident light. The spectrum ranging from the visible to the near-infrared band (0.36 μm–2.5 μm) was measured using the Cary 7000 UV-Vis-NIR spectrophotometers, with a resolution of 0.1 nm. The morphology of the metasurface was examined using the X-Max 20 scanning electron microscope at 5 kV. The 3D morphology was obtained by scanning with the confocal microscopy (VK-X3000).

### 3.4 Protein preparation and multipeak fitting

The BSA protein from Sigma-Aldrich was diluted in deionized water using magnetic stirring to obtain a solution with a concentration of 100 mg/mL. A series of BSA solutions with different concentrations (0, 0.2, 0.5, 1, 2 mg/mL) were prepared. Subsequently, a 10 μL droplet of the BSA solution was deposited onto the metasurface. BSA concentrations below the nanomolar level (0, 0.03, 0.075, 0.15, 0.3 nmol) were applied to the entire transducer surface, considering the molecular mass of BSA (66,430 g/mol). Finally, the samples were carefully dried using a nitrogen blow to remove any water absorption. The distribution of BSA molecules on the 1.5 cm^2^ sample surface was assumed to be homogeneous. Since the light spots of the FTIR have an area of approximately 0.5 cm^2^, the theoretically detectable minimum amount of BSA is 10 pmol. The 0 μg measurement corresponds to drying ultrapure water on the metasurface. The measurement with a series mass protein deposition is conduct on the same metasurface. The metasurface is sock and cleaned in ultrapure water. The infrared spectrum of metasurface is tested to ensure complete cleanliness of the metasurface before protein deposition.

The secondary derivative is the most common used to separate overlapping bands. One major advantages of SD analysis are that it can be performed objectively without the need for arbitrary selection of deconvolution parameters. In determining the secondary structure of proteins, the second derivative spectrum was calculated using a seven-point Savitsky–Golay second-derivative function. The selection of a seven-point calculation window is based on the fact that 14 cm^−1^ resolution is typically used to collect protein spectra. The 14 cm^−1^ spectral region is narrower than the half-bandwidth at half-height for most amide I band components of various secondary-structural elements, thereby minimizing distortion in the amide I band components. The peak frequency in the secondary derivative was identical to the original peak frequency, while the half-width of the second derivative was related to the half-width of the original line (*K* = 2.7) [52].

The spectrum of the metasurface with BSA coating under S and P polarization can be fitted with a Lorentz model with two and three coupled oscillators, respectively. The Multipeak fitting program (Origin 2017) iterated the curve-fitting with the least squares estimation starting from initial values. The initial values for amide absorption were set based on the wavelength and full width at half maximum (FWHM) of the amide peaks in the experimental reflection spectrum, and the FWHM of the resonance was fixed according to the spectrum of the blank metasurface. These fixed parameters were summarized in Supplementary Table 1. Each iteration reached convergence, and each squared of curve fitting was above 0.95. The band area for amide absorption and metasurface resonance was used to calculate the relative contribution of each component to the spectrum. The characteristics of the band were represented as follows: (i) the area of the amide peak represents its absorption, and (ii) the wavelength of the resonance.

## 4 Conclusions

In this study, we present a dual resonant metasurface that utilizes a metal–dielectric–metal plasmonic grating and a dielectric–metal channel. By adjusting the vertical and horizontal structures of metasurface, we can independently modify the spectrum of the metasurfaces in the near-infrared and mid-infrared regions. This broadband infrared metasurface exhibits robust spectral regulation, enabling a polarization-dependent dual-resonance strategy that offers a significant competitive advantage over traditional metallic nanostructure in refractive index sensing in the near-infrared and ultrasensitive vibrational spectroscopy in the mid-infrared. Specifically, our proposed metasurface achieves protein concentration sensing and dynamic monitoring of protein concentration in the infrared two-zone. Additionally, it enhances the mid-infrared absorption of amide II with a high *Q* resonance. This metasurface, which combines wavelength multiplexing and polarization dependence for protein recognition and trace detection, presents a novel approach for developing high-performance sensors and other photonics applications in the broadband infrared region.

## Supplementary Material

Supplementary Material Details
