# Supplementary material for: Wavelength multiplexing infrared metasurfaces for protein recognition and trace detection
Source: Nanophotonics. 2023 Sep 26;12(20):3963–76. doi: 10.1515/nanoph-2023-0517 (PMC11501136; doi:10.1515/nanoph-2023-0517)
Supplement: Supplementary file 1 — Supplementary Material Details [file j_nanoph-2023-0517_suppl_001.docx]

Supporting Information

**Wavelength multiplexing infrared metasurfaces for protein recognition and trace detection**

Shiqing Dong ^1^, Chao Dong ^2^, Kesheng Shen ^1^, Yunzheng ^1^, Jie Sun ^1^, Cheng Zhen ^1^, Haiyang Hu ^1^, Feng Zhang ^1^, Zhe Zhang ^1^, Hongchao Liu ^1, *^ and Hai Lu ^1, *^

^1^ Engineering Laboratory for Optoelectronic Technology and Advanced Manufacturing, School of Physics, Henan Normal University, Xinxiang 453007, China;

^2^ Henan Key Laboratory of Optoelectronic Sensing Integrated Application, College of Electronic and Electrical Engineering, Henan Normal University, Xinxiang 453007, China.

1. **numerical design of metasurfaces**


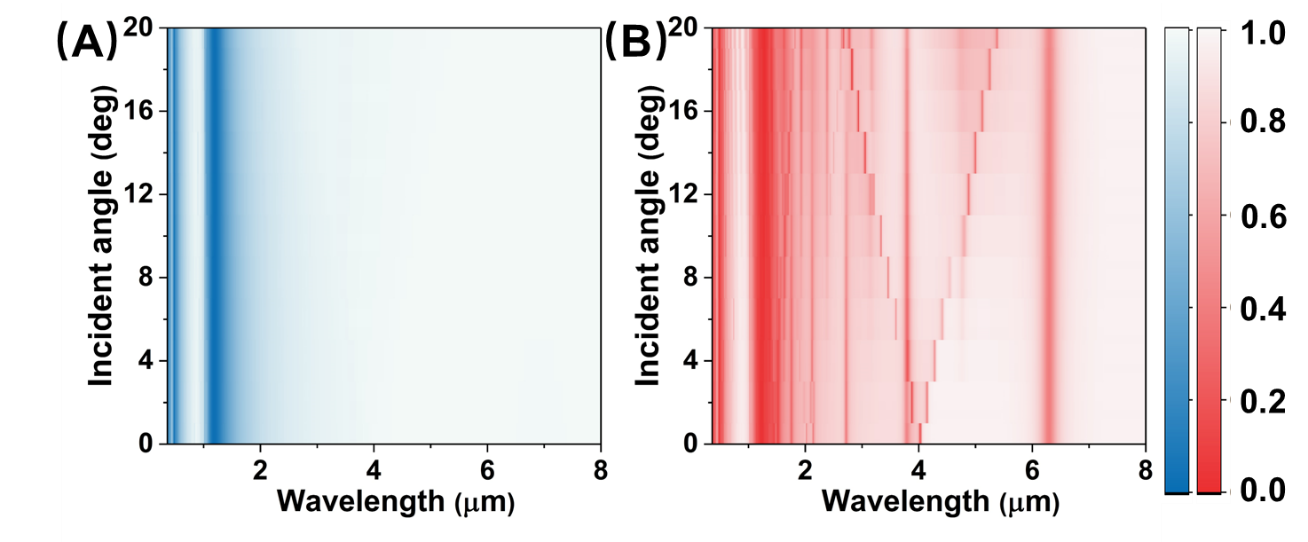


**Figure S1.** Dependence of the numerical reflection spectra on different incident angle under the S polarization (A) and the P polarization (B).


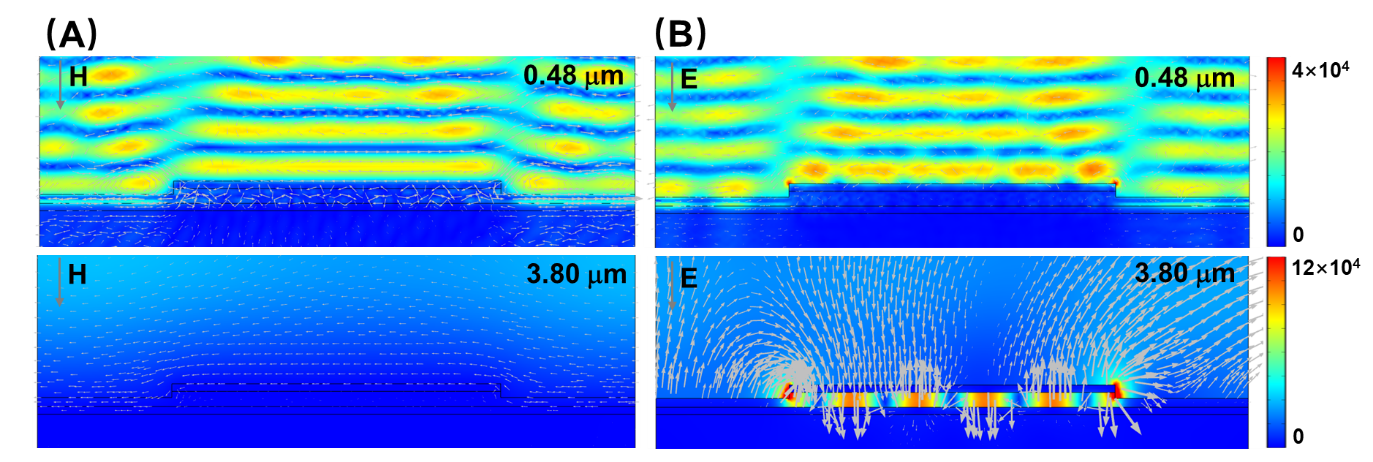


**Figure S2.** (A) The electric field distributions and the magnetic vector of S polarization with 6 degree incidence in the xy plane. (B) The electric field distributions and electric vector of P polarization with 6 degree incidence.


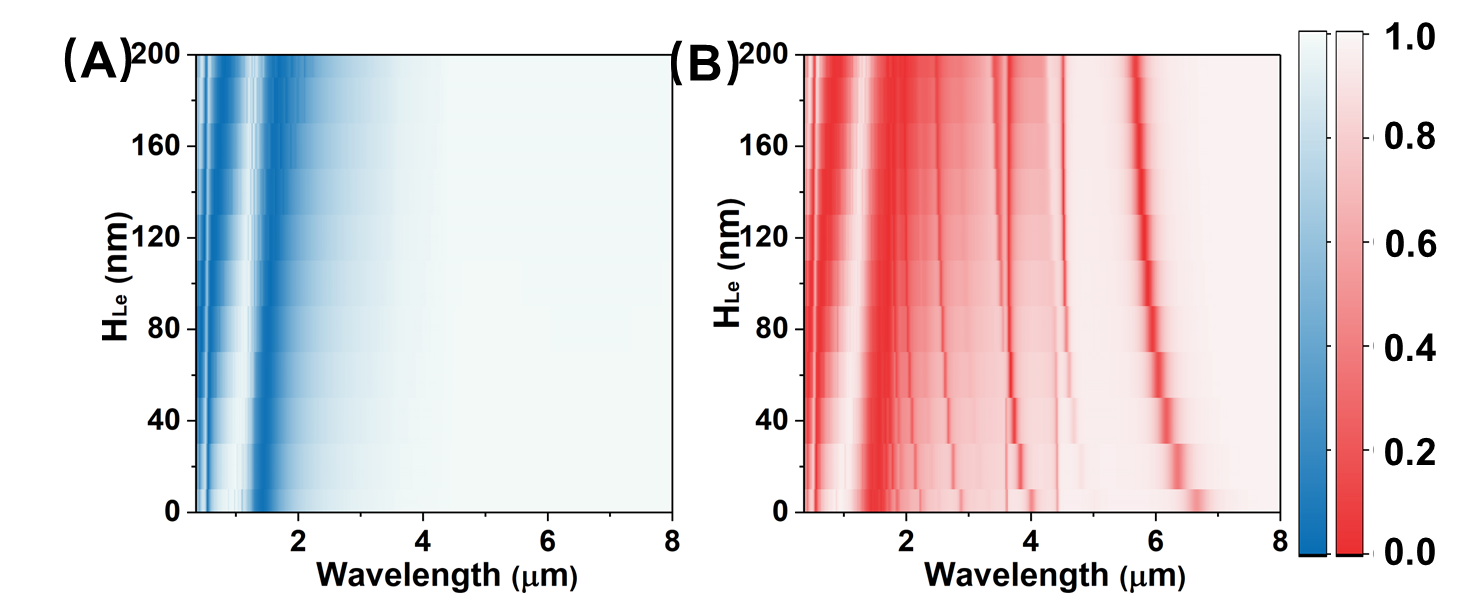


**Figure S3.** Function of the numerical reflection spectra on H_Le_ under the S polarization (A) and the P polarization (B).

1. **morphology characterization of metasurface**


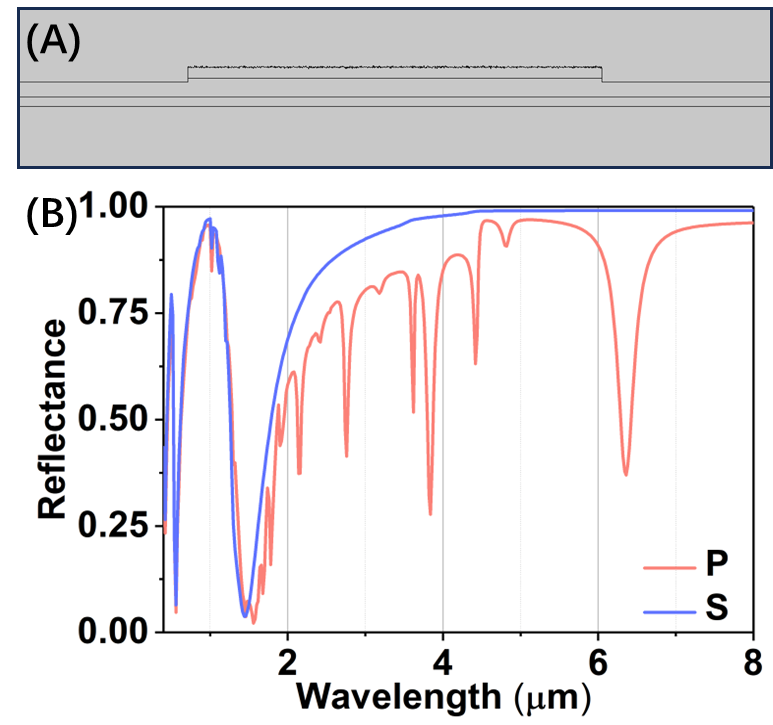


**Figure S4.** The effect of inhomogeneity metasurface on spectra with different polarization. (A) The numerical model of inhomogeneity metasurface. (B) The spectra of inhomogeneity metasurface with different polarization.


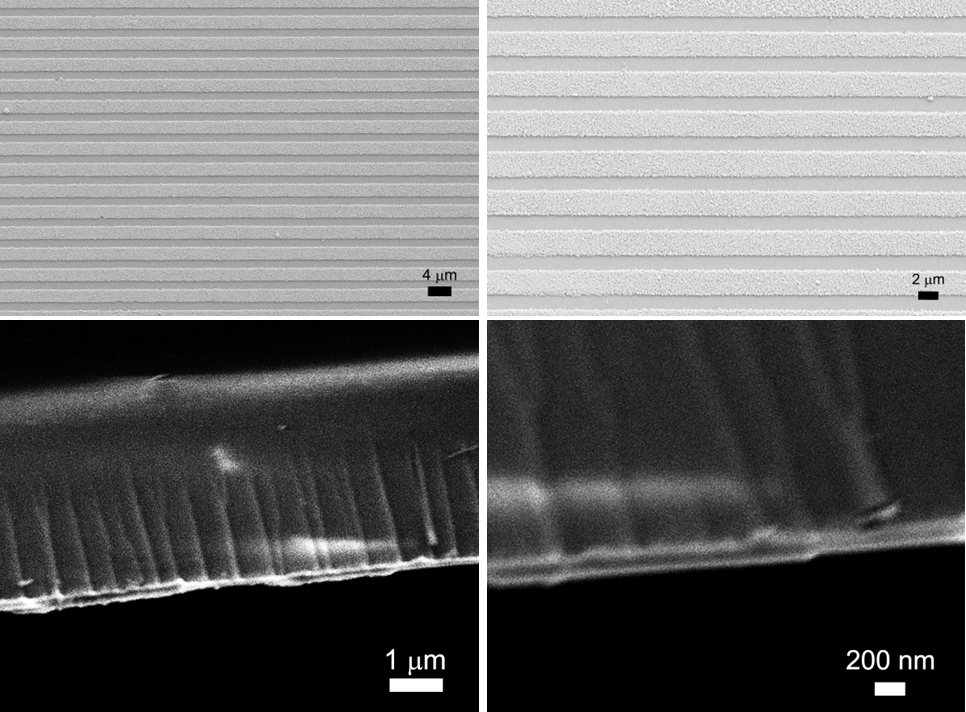


**Figure S5.** the top view and cutaway of metasurface with SEM.


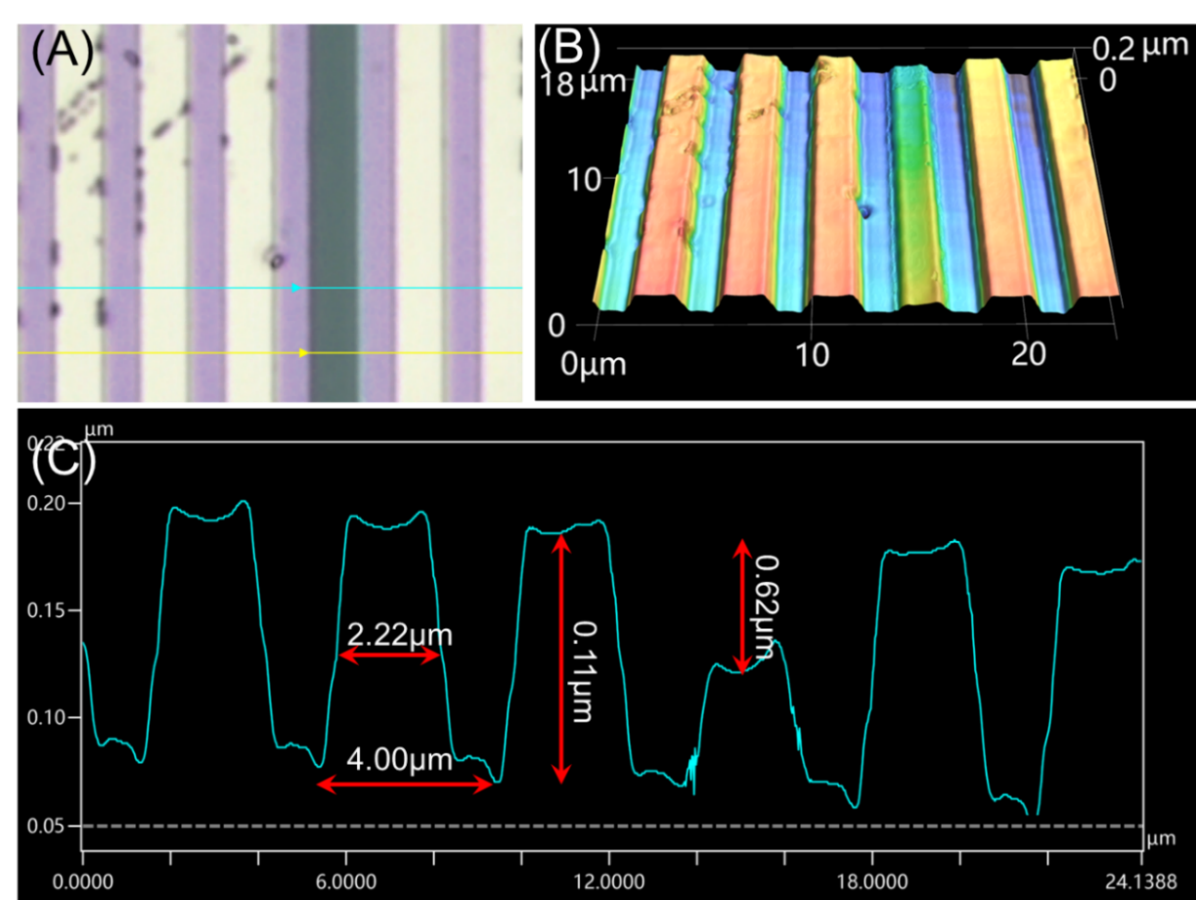


**Figure S6.** The metasurface morphology of confocal microscope. (A) the confocal image of metasurface. (B) The 3D modeling of metasurface gratings, the vertical structure shown in the diagram is ten times larger than its original size. (C) The height analysis of the blue line.

1. **Lorentz fitting for the protein sensing**


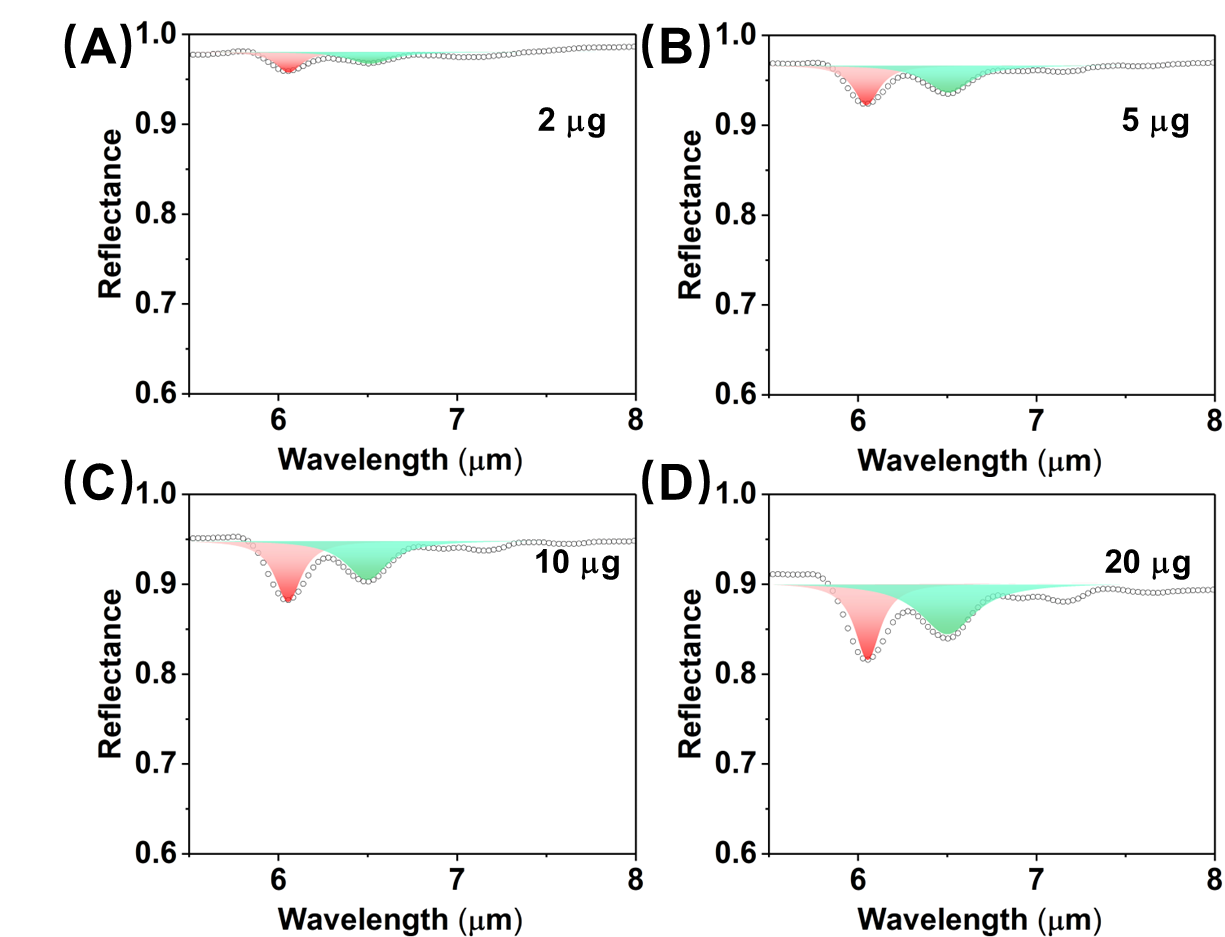


**Figure S6.** The multipeak fitting of reflection spectra with different mass BSA coating under S polarization (A. 2 μg, B. 5 μg, C. 10 μg, D. 20 μg). The red, green and blue peaks represent the Lorentz peaks of amide I, amide II and metasurface resonance, respectively.


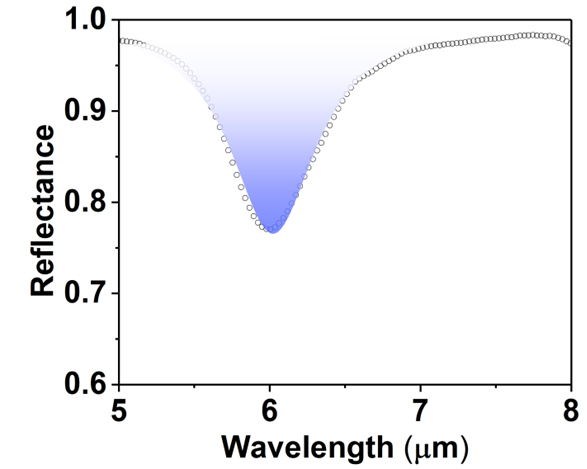


**Figure S7.** Reflectance and single Lorentz fitting of metasurface under P polarization.

**Supplement Tab. 1:** The Lorentz Fitting parameters of spectrum under S polarization.

| Deposition mass | Centre amide I | FWHM amide I | Centre amide II | FWHM amide II |
| --- | --- | --- | --- | --- |
| 2 μg | 6.058 μm | 0.175 μm | 6.511 μm | 0.295 μm |
| 5 μg | 6.047 μm | 0.179 μm | 6.506 μm | 0.300 μm |
| 10 μg | 6.054 μm | 0.181 μm | 6.497 μm | 0.287 μm |
| 20 μg | 6.054 μm | 0.176 μm | 6.499 μm | 0.376 μm |

**Supplement Tab. 2:** The Lorentz Fitting parameters of spectrum under P polarization.

| Deposition mass | Area amide I  S polarization | Area amide II  S polarization | Area amide I  P polarization | Area amide II  P polarization | Wavelength of resonance |
| --- | --- | --- | --- | --- | --- |
| 2 μg | -0.01491 | -0.01322 | -0.03594 | -0.04808 | 6.789 μm |
| 5 μg | -0.03374 | -0.03041 | -0.07814 | -0.09092 | 6.821 μm |
| 10 μg | -0.06421 | -0.05713 | -0.10629 | -0.11494 | 6.890 μm |
| 20 μg | -0.12352 | -0.10834 | -0.16091 | -0.17899 | 7.147 μm |
